# The Conduct of the War in the Crimea

**Published:** 1855-09

**Authors:** 


### THE CONDUCT OF THE WAR IN THE CRIMEA.

Mr. Roebuck and his Committee in their enquiries into the conduct of the war, overhaul medical as well as military delinquents. We clip the following specimen of the manner in which Mr. Roebuck handles witnesses from the New York Evening Post:

"Was to day at the Committee of Inquiry, Mr. Roebuck's committee. The proceedings are entirely public. To-day two of the Crimean medical officers were on the rack, and pretty disclosures they were forced to make, more to the edification of their hearers than to their own delight, for they proved themselves to have been guilty of the grossest negligence in their offices. One of them was the inspector-general or deputy inspector of the hospital at Scutari, whose duty it was to go about and see that everything connected with this department was in a proper state—and imagine this man saying that the hospitals were in a cleanly condition, and writing to Lord Stratford de Redcliffe that they had everything needful for the comfort and sustenance of the patients, at the very time that the hospitals were in the most disgusting and filthy condition, and men dying in them of mere exhaustion. The Committee pumped them both dry in a manner which was delightful. They both hemmed and hawed, and tried to get out of the mud the best way they could; but little Roebuck put it into them in a way they hadn't been accustomed to before, snubbing them at a most prodigious rate. 'Why can't you answer that question in a plain manner? Did you, or did you not?' 'No.' 'Well, then, say so at once.'"

